# Unintended changes in ocular biometric parameters during a 6-month follow-up period after FS-LASIK and SMILE

**DOI:** 10.1186/s40662-021-00232-8

**Published:** 2021-03-19

**Authors:** Junjie Wang, Bernardo T. Lopes, Hechen Li, Riccardo Vinciguerra, Si Cao, Songan Wu, Rong Zhu, Qinmei Wang, Xiaobo Zheng, Fangjun Bao, Ahmed Elsheikh

**Affiliations:** 1 Eye Hospital, Wenzhou Medical University, Wenzhou, 325027 Zhejiang China; 2 The Institution of Ocular Biomechanics, Wenzhou Medical University, Wenzhou, 325027 Zhejiang China; 3 School of Engineering, University of Liverpool, Liverpool, L69 3GH UK; 4 Humanitas San Pio X Hospital, Milan, Italy; 5 National Institute for Health Research (NIHR) Biomedical Research Centre for Ophthalmology, Moorfields Eye Hospital NHS Foundation Trust and UCL Institute of Ophthalmology, London, UK; 6 Beijing Advanced Innovation Center for Biomedical Engineering, Beihang University, Beijing, China

**Keywords:** Posterior corneal surface, Internal anterior chamber depth, The length from corneal endothelium to retina, FS-LASIK, SMILE

## Abstract

**Background:**

Corneal refractive surgery has become reliable for correcting refractive errors, but it can induce unintended ocular changes that alter refractive outcomes. This study is to evaluate the unintended changes in ocular biometric parameters over a 6-month follow-up period after femtosecond laser-assisted laser in situ keratomileusis (FS-LASIK) and small incision lenticule extraction (SMILE).

**Methods:**

156 consecutive myopic patients scheduled for FS-LASIK and SMILE were included in this study. Central corneal thickness (CCT), mean curvature of the corneal posterior surface (K_pm_), internal anterior chamber depth (IACD) and the length from corneal endothelium to retina (ER) were evaluated before and after surgery over a 6-month period.

**Results:**

Both the FS-LASIK and SMILE groups (closely matched at the pre-surgery stage) experienced flatter K_pm_, shallower IACD and decreased ER 1 week post-surgery (*P* < 0.01), and these changes were larger in FS-LASIK than in SMILE group. During the 1 week to 6 months follow up period, K_pm_, IACD and ER remained stable unlike CCT which increased significantly (*P* < 0.05), more in the FS-LASIK group.

**Conclusions:**

During the follow up, the posterior corneal surface became flatter and shifted posteriorly, the anterior chamber depth and the length from the corneal endothelium to retina decreased significantly compared with the pre-surgery stage. These unintended changes in ocular biometric parameters were greater in patients undergoing FS-LASIK than SMILE. The changes present clear challenges for IOL power calculations and should be considered to avoid affecting the outcome of cataract surgery.

## Background

In corneal laser vision correction surgery for myopic patients, the cornea is reshaped with the aim to reach emmetropia. One way to achieve the desired reshaping is using excimer laser to ablate the anterior corneal tissue with or without a flap, in laser in situ keratomileusis (LASIK) or photorefractive keratectomy (PRK), respectively [1]. Another way is by removing a stromal lenticule underneath a corneal cap in a procedure called small incision lenticule extraction (SMILE) [2].

In these procedures, most of the corneal reshaping takes place in the anterior surface, which is directly affected by the surgery and becomes flattened, compensating the imbalance between corneal curvature and axial length that exists in myopic patients [3]. However, changes in the posterior surface have been reported in the literature for these three procedures [4, 5]. These posterior changes impact the calculation of the keratometric index of refraction, reducing the accuracy of the intraocular lens power calculation [6]. The detection of iatrogenic ectasia whose early diagnosis relies on the posterior surface topography is also affected by these post-surgery changes [7].

Changes in corneal posterior shape and ocular biometric parameters are unintended and result from surgical procedures that are only planned to affect the anterior corneal surface. This study aims to assess the indirect effects of femtosecond laser-assisted laser in situ keratomileusis (FS-LASIK) and SMILE on the corneal posterior shape through measurement of ocular biometric parameters that can influence the overall refractive power and the satisfaction of patients post refractive surgeries.

## Methods

### Patients

This retrospective comparative cases series was approved by the Ethics Committee of the Eye Hospital, Wenzhou Medical University (2019–002-K-02). Medical records of patients that underwent refractive surgery by either FS-LASIK or SMILE methods from October 2016 to September 2018 were reviewed. The inclusion criteria were the presence of myopia accompanied or not by astigmatism of less than 3.25 D, with resulting manifest spherical equivalent (MSE) not below −10.00 D, minimum age of 18 years old, absence of ocular diseases other than refractive errors and no records of complications during or after the procedure. All patients underwent complete ophthalmic examination and those soft contact lens wearers were asked to stop the use of the lens for 2 weeks. From the 150 cases selected, 77 patients underwent FS-LASIK surgery and 73 received SMILE surgery. Patients from each group were further subdivided according to the MSE, into low to moderate myopia group (LM), MSE ≥ −5.00D (FS-LASIK 26 eyes, SMILE 33 eyes) and high myopia group (HM), MSE < −5.00D (FS-LASIK 51 eyes, SMILE 40 eyes).

In the FS-LASIK procedure, 100 to 110 μm thick flap with a superior 45°-wide hinge was created using a femtosecond IntraLase IFS150 laser machine (Abbott Medical Optics, CA, USA). This step was followed by tissue ablation using an Amaris 750 excimer laser (Schwind eye-tech-solutions, Kleinostheim, Germany). SMILE was performed using the VisuMax femtosecond laser system (Carl Zeiss Meditec AG, Jena, Germany), and the laser settings were as follows: 120 μm intended cap thickness, 6.0 to 6.9 mm optical zone (lenticule diameter), and a 2-mm side cut at the 10–0’clock position. Refractive error correction (REC) and optical zone diameter (OZD) for FS-LASIK and SMILE were recorded.

### Measurements and data

**Fig. 1:**
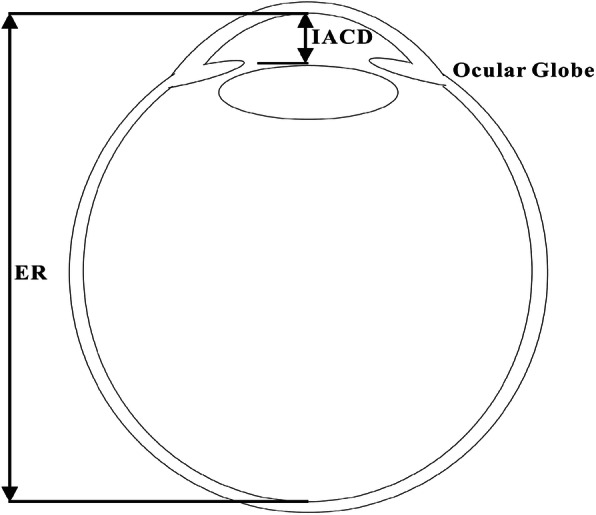
Sketch of ocular globe showing main biometric dimensions including internal anterior chamber depth (IACD) and distance from corneal endothelium to retina, ER

The Lenstar LS 900 (Hagg-Streit AG, Koeniz, Switzerland), a non-contact biometry device, was employed in this study to simultaneously image the cornea, internal anterior chamber, central crystalline lens and fovea. The device uses an optical low coherence reflectometry (OLCR) technology that has a broad-band light source (20–30 nm) with a central wavelength of 820 mm. The repeatability of the Lenstar’s axial biometric parameter measurements was reported to be excellent, precision of axial length was 0.02 ~ 0.03 mm in axial length measurement of normal eyes [8, 9], and the within-subject standard deviation (Sw) was 2.9 μm [10] in central cornea thickness (CCT) measurement after LASIK. Measurements with the Lenstar included CCT, internal anterior chamber depth (IACD) and the distance from the corneal endothelium to the retina (ER); see Fig. 1.

The mean curvature of corneal posterior surface (K_pm_, the mean of curvatures in horizontal and vertical directions over the central 3 mm diameter zone) was provided by a Pentacam (OCULUS Optikgerate GmbH, Wetzlar, Germany). Manifest refractive error was measured with a phoroptor (Nidek RT-2100; Nidek Inc., Gamagori, Japan). The Dynamic Contour Tonometer (DCT, SMT Swiss Microtechnology AG, Switzerland) was employed in this study for intraocular pressure measurement (IOP). Two experienced examiners (SC and HCL) performed all scans in 30-min sessions held between 09:00 and 17:00. All measurements were taken before surgery (pre), and 1 week (pos1w), 1 month (pos1m), 3 months (pos3m) and 6 months (pos6m) after surgery. The wound healing effect was expected to have stabilized around 6 months after the surgery and therefore this time period was used in follow up in earlier studies [11, 12]. Three consecutive Pentacam and Lenstar measurements were taken per sitting for both eyes in a dimly lit room without pupillary dilation, and the mean of the three measurements taken for each right eye was used in the statistical analysis.

### Statistical analysis

All analyses were performed using the PASW Statistics 20.0 (SPSS Inc., Chicago, USA). After confirming the presence of normal distribution in the studied groups, comparisons of age, IOP, REC and OZD between the two surgery groups for different MSE subgroups were performed using the One-way ANOVA in each subgroup, while the comparison of MSE, CCT, K_pm,_ IACD and ER in the LASIK and SMILE surgery groups in different pre and postoperative periods were performed using the MANOVA of repeated measurements. Multiple linear regression analyses with the stepwise method were used to identify significant associations of CCT, REC and OZD with the K_pm_ differences between pre and post-operation stages. *P* values less than 0.05 were considered indicative of statistical significance.

## Results

No significant difference between FS-LASIK and SMILE groups was observed in baseline parameters: age, gender, preoperative IOP, REC, OZD, K_pm_, CCT and the biometric IACD and ER (*P* > 0.05). The demographic statistics are summarized in Table 1 and the biometric parameters’ baseline values and variations during follow-up are shown in Table 2.

**Table 1:** Matched demographics for the two surgery groups

Biometric parameter	Subgroups	FS-LASIK	SMILE	P
Age (years)	LM group	26.93 ± 5.17	26.09 ± 5.92	0.567
	HM group	26.41 ± 4.73	26.50 ± 4.52	0.930
Gender ratio	LM group	10/16	17/16	0.431
	HM group	24/27	17/23	0.678
IOP (mmHg)	LM group	17.63 ± 1.29	18.04 ± 3.07	0.146
	HM group	18.03 ± 2.37	18.43 ± 2.14	0.509
REC (D)	LM group	−3.85 ± 0.87	−3.97 ± 0.74	0.708
	HM group	−6.39 ± 0.79	−6.71 ± 1.14	0.230
OZD (mm)	LM group	6.80 ± 0.28	6.71 ± 0.09	0.114
	HM group	6.57 ± 0.25	6.49 ± 0.25	0.095

*FS-LASIK=* femtosecond laser-assisted laser in situ keratomileusis, *SMILE=* small incision lenticule extraction; gender ratio = male/female, *IOP=* intraocular pressure measurement by DCT, *REC=* refractive error correction, *OZD=* optical zone diameter, *LM= group* low to moderate myopia group, *HM= group* high myopia group

**Table 2:** Ocular biometric parameters before and after corneal refractive surgery

Variable	Refractive Status	Surgery Group	pre	pos1w	pos1m	pos3m	pos6m	pre vs pos1w	pos1w vs pos6m
MSE (D)	LM	FS-LASIK	−3.85 ± 0.87	0.29 ± 0.30	0.18 ± 0.38	0.11 ± 0.28	0.22 ± 0.29	0.000	1.000
		SMILE	−3.97 ± 0.74	0.23 ± 0.39	0.25 ± 0.39	0.28 ± 0.35	0.23 ± 0.3	0.000	1.000
	HM	FS-LASIK	−6.39 ± 0.79	0.25 ± 0.43	0.21 ± 0.41	0.13 ± 0.50	0.11 ± 0.45	0.000	0.099
		SMILE	−6.71 ± 1.14	0.01 ± 0.47	0.03 ± 0.47	−0.02 ± 0.4	0.05 ± 0.38	0.000	1.000
Kpm (D)	LM	FS-LASIK	−6.31 ± 0.17	−6.30 ± 0.19	−6.29 ± 0.18	−6.29 ± 0.17	−6.30 ± 0.18	0.911	1.000
		SMILE	−6.20 ± 0.28	−6.19 ± 0.28	−6.20 ± 0.26	−6.20 ± 0.28	−6.19 ± 0.27	1.000	1.000
	HM	FS-LASIK	−6.27 ± 0.21	−6.24 ± 0.21	−6.23 ± 0.21	−6.24 ± 0.22	−6.23 ± 0.20	0.000	1.000
		SMILE	−6.32 ± 0.25	−6.32 ± 0.25	−6.31 ± 0.25	−6.30 ± 0.25	−6.30 ± 0.25	0.561	1.000
CCT (μm)	LM	FS-LASIK	545.08 ± 24.21	465.92 ± 36.71	469.77 ± 36.68	473.88 ± 37.11	474.85 ± 38.38	0.000	0.000
		SMILE	545.48 ± 20.34	464.21 ± 19.93	462.58 ± 19.38	467.15 ± 18.03	467.88 ± 19.6	0.000	0.072
	HM	FS-LASIK	548.94 ± 32.66	434.49 ± 34.3	438.12 ± 34.99	443.53 ± 34.02	446.08 ± 33.94	0.000	0.000
		SMILE	552.65 ± 19.97	440.40 ± 20.09	442.15 ± 19.17	447.53 ± 18.49	448.53 ± 18.61	0.000	0.000
IACD (mm)	LM	FS-LASIK	3.14 ± 0.27	3.05 ± 0.27	3.07 ± 0.29	3.06 ± 0.29	3.05 ± 0.28	0.000	1.000
		SMILE	3.15 ± 0.22	3.07 ± 0.23	3.08 ± 0.22	3.08 ± 0.23	3.07 ± 0.21	0.000	1.000
	HM	FS-LASIK	3.21 ± 0.25	3.11 ± 0.26	3.14 ± 0.26	3.15 ± 0.26	3.13 ± 0.26	0.000	0.100
		SMILE	3.19 ± 0.21	3.13 ± 0.21	3.13 ± 0.21	3.13 ± 0.20	3.14 ± 0.20	0.000	1.000
ER (mm)	LM	FS-LASIK	24.57 ± 0.71	24.54 ± 0.71	24.55 ± 0.71	24.54 ± 0.72	24.53 ± 0.73	0.000	1.000
		SMILE	24.98 ± 0.88	24.96 ± 0.88	24.95 ± 0.89	24.95 ± 0.89	24.96 ± 0.89	0.047	1.000
	HM	FS-LASIK	25.69 ± 0.79	25.68 ± 0.79	25.68 ± 0.79	25.69 ± 0.79	25.7 ± 0.79	0.065	0.585
		SMILE	25.67 ± 0.99	25.67 ± 0.99	25.64 ± 0.99	25.62 ± 0.98	25.62 ± 0.98	0.000	1.000

*MSE=* spherical equivalent refraction, *K*_*pm*_ = mean corneal curvature of posterior surface, *CCT=* central corneal thickness, *IACD=* internal anterior chamber, *ER=* distance from corneal endothelium to retina, *LM= group* low to moderate myopia group, *HM= group* high myopia group

The longitudinal analysis showed different behaviors between FS-LASIK and SMILE in the MSE. While both LM and HM subgroups presented postop stable MSE over 6 m after SMILE, the MSE of patients that underwent FS-LASIK reduced significantly from 1w to 1 m (*P* < 0.05) and then became stable until the 6th month (*P* > 0.05). The comparison of MSE values between the procedures was only significant at the first week in the HM group (*P <* 0.05).

Regarding CCT, after the initial reduction after both procedures, there was a gradual increase until the end of the follow-up period. This increase was higher in FS-LASIK than in SMILE in both LM and HM subgroups. Considering the period between the first week and the 6th month post-surgery, the LM subgroup presented increases in CCT of 8.9 ± 6.3 μm in FS-LASIK and 3.7 ± 8.3 μm in SMILE, while in the HM subgroup the increase was 11.6 ± 6.6 μm in FS-LASIK and 8.1 ± 9.3 in SMILE.

**Fig. 2:**
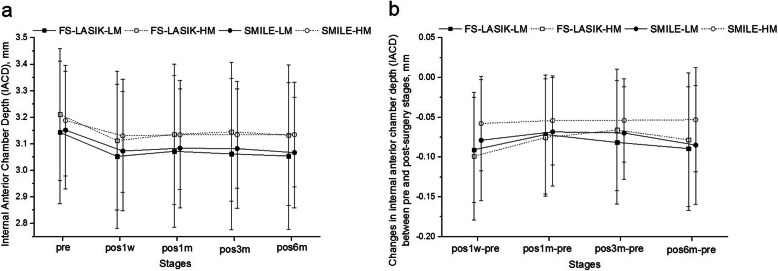
Primal value (**a**) and change (**b**) in internal anterior chamber depth (IACD) pre-operation and at different stages post FS-LASIK and SMILE. FS-LASIK = femtosecond laser-assisted laser in situ keratomileusis, SMILE = small incision lenticule extraction, pos1w, pos1m, pos3m and pos6m mean 1 week, 1 month, 3 months and 6 months post-operation, respectively; LM = low to moderate myopia group, HM = high myopia group

**Fig. 3:**
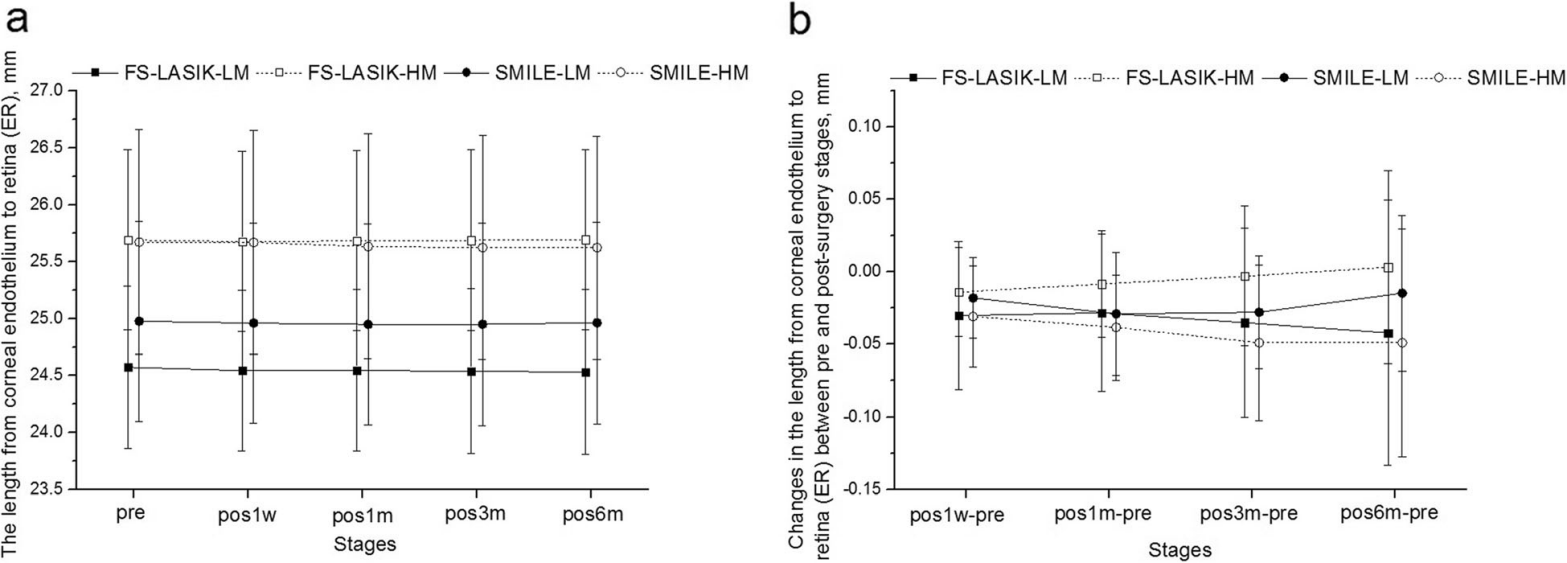
Primal value (**a**) and change (**b**) in the length from corneal endothelium to retina (ER) pre-operation and at different stages post FS-LASIK and SMILE. FS-LASIK = femtosecond laser-assisted laser in situ keratomileusis, SMILE = small incision lenticule extraction, pos1w, pos1m, pos3m and pos6m mean 1 week, 1 month, 3 months and 6 months post-operation; LM = low to moderate myopia group, HM = high myopia group

A slight flattening (increase in the negative posterior curvature) was observed 1 week after each procedure, and that flattening remained stable thereafter. The changes in K_pm_ between pre and pos1w were higher (*P* < 0.01) in the FS-LASIK group (0.03 ± 0.03 D, 0.49 ± 0.55%) than in the SMILE group (0.01 ± 0.03 D, 0.16 ± 0.53%). The K_pm_ were also higher (*P <* 0.01) in both the HM subgroups than in the LM subgroups (0.03 ± 0.04 D, 0.40 ± 0.56% vs 0.01 ± 0.03 D, 0.19 ± 0.55%). No further significant change in posterior curvature was observed within the rest of the follow-up period (*P* > 0.05) in both groups. The results also showed significant reductions in IACD 1 week after surgery (*P* < 0.05) in both procedures. Over the rest of the follow-up, the two surgery groups differed with steady changes in IACD in the SMILE group compared to slight fluctuations in the FS-LASIK group; see Fig. 2. The reduction in IACD from pre to pos1w was higher in the FS-LASIK group than in the SMILE group (−0.096 ± 0.075 mm vs −0.067 ± 0.068 mm) (*P <* 0.05), while was similar (*P* = 0.788) between the HM and LM subgroups. ER decreased 1 week after surgery (by − 0.019 ± 0.039 mm in FS-LASIK and − 0.025 ± 0.032 mm in SMILE) compared with pre surgery stage (*P* < 0.01), then remained stable thereafter; see Fig. 3. The change in ER between pre and pos1w was similar among the two surgery groups (*P* = 0.354), and no significant change was observed between the HM and LM subgroups (*P* = 0.728).

Table 3 summarizes the results of multiple linear regression analyses in the study group. The analysis shows that only the REC and OZD were correlated with the difference in K_pm_ between pre and pos6m stages.

**Table 3:** Stepwise multiple linear regression models for IOP differences between pre and post-operation stage

Dependent Variables	Parameters	Β ^a^	*P* value	Regression Equation	Adjusted R^2^	F ^b^	*P* value
∆K_pm-prepos6m_	REC	−0.282	0.003	∆K_pm-prepos6m_ = −0.007 x REC(D) + 0.030 x OZD (mm) – 0.214 (D)	0.251	4.783	0.010
	OZD	0.200	0.034				

∆ means the difference between pre and post operation stages; prepos6m means the difference between pre and pos6m stages; K_pm_ means mean curvature of the corneal posterior surface

^a^means standardized coefficients (Beta)

^b^means multiple linear regression model (Stepwise)

## Discussion

Corneal refractive surgeries are conceptually designed to correct refractive errors through reshaping the anterior surface, which accounts for most of the corneal refractive power. However, since the surgical procedures affect corneal biomechanics (through tissue separation, ablation and triggering of wound healing), the cornea may experience additional deformation under the same IOP, causing shape changes in the posterior surface. This study aimed to characterize these unintended changes that play an important role in the surgical outcome through analysis of clinical data obtained before and after FS-LASIK and SMILE surgeries.

The main results of this study indicated that:

- The cornea became thicker during follow up after both surgeries;
- The posterior corneal surface became slightly flatter with a posterior shift;
- The anterior chamber depth decreased significantly;
- These effects were lower in low myopia patients than in high myopia patients;
- The effects were larger and more consistent in FS-LASIK than in SMILE.

Up to 6 months follow-up was included in this study, which enabled analysis of the mid-term shape changes following both refractive surgeries and the subsequent wound healing process. For the first main result, thickness measurements, the immediate reductions caused by ablation was followed by slight increases over the 6 months follow up period which is expected due to epithelial thickening at the center of the cornea due to the myopic ablation [13]. However, the increase in corneal thickness was significantly higher in the FS-LASIK than in the SMILE group (10.7 ± 6.6 μm vs 6.1 ± 9.1 μm), and larger in the high myopia group than low to moderate myopia group (10.1 ± 8.0 μm vs 6.0 ± 7.9 μm), although the difference might not be clinically relevant. This could be due to a difference in the gradient of the corneal curvature that is known to drive shape changes and epithelial remodeling after refractive surgery [14, 15]. Similarly, Ryu et al’s study reported postoperative changes in epithelial thickness that were larger after FS-LASIK surgery than after SMILE [16]. Additionally, Reinstein et al. observed that the difference between the planned tissue removal and the experienced stromal reduction was 8.2 ± 8.0 μm. It was hypothesized that there is stromal expansion after SMILE which could be at least partially compensated by the lower epithelial thickening [17]. This study, however, did not include segmental tomography analysis precluding the ability to perform a separate analysis of epithelial and stromal thicknesses. Therefore, it was not possible to determine if the thickening effect post-surgery has taken place in the epithelium, stroma or both.

The second main result, regarding the mild posterior surface flattening was only significant at the first week postoperative and was higher in the FS-LASIK and in the HM group. After this initial flattening, the posterior cornea remained stable in a slightly flatter shape. The level of flattening was correlated with refractive error correction and optical zone diameter. Dupps and Roberts have also observed posterior flattening in an ex vivo study and proposed a biomechanical mechanism for this finding [18]. The variation in correlation between the anterior and posterior ocular surfaces after FS-LASIK and SMILE could result in a difference in corneal refraction index, which if ignored may induce unexpected outcomes for IOL. For this reason, it was suggested to use individualized biometrical IOL formulas in the IOL calculations needed when performing cataract surgery in eyes that have been through corneal refractive surgery [19].

In most of the previous clinical studies, the posterior surface was expressed in terms of its elevation relative to a reference surface. In this study, we chose not to rely on the relative elevation as the downward shift of corneal apex caused by corneal ablation introduces changes in the coordinate system used post-surgery and may therefore affect the results. Besides, the region over which the reference surface calculation is conducted – commonly the central 8–9 mm diameter area – does not remain stable after the surgery procedure [20–22]. This effect leads to variations in the reference surface (such as the best-fit sphere, BFS) post-surgery, relative to that used pre-surgery, possibly causing further measurement inaccuracies [5, 20]. For these reasons, curvature, which depends on the relative position of adjacent points and is not influenced by the change in reference plane was used in this study to allow a more realistic appreciation of corneal behavior and the result observed with this strategy was in accordance to the expected change in biomechanical behavior caused by the surgery.

Stronger consistency than the results reported in earlier studies was found in our measurements of other parameters. While earlier studies reported mean reductions in IACD of 0.04 mm at 1 month after surgery [23], 0.02 ± 0.07 mm after 2 months [24] and 0.06 ± 0.05 mm after 6 months [25], our study found significant reductions in IACD at 1 month, 3 months and 6 months compared with pre-operative values of 0.067 ± 0.068 mm, 0.066 ± 0.067 mm, 0.075 ± 0.077 mm, respectively, *P* < 0.01. This difference can at least be partly due to a backward shift of the posterior corneal surface.

**Fig. 4:**
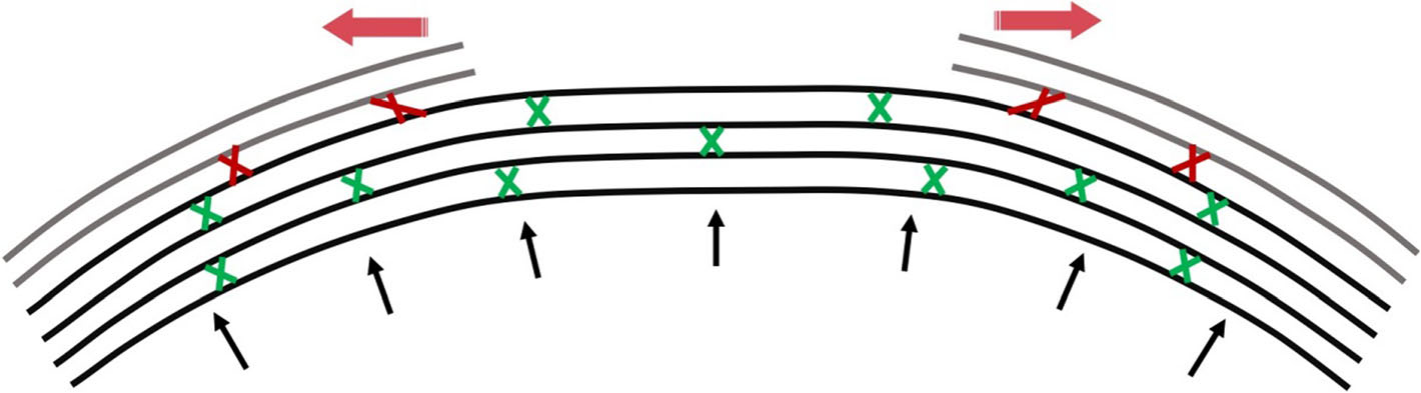
Change in the posterior corneal surface because of biomechanical alternations in corneal structure caused by refractive surgery

Interestingly, the length from the endothelium to the retina’s front surface (ER or axial length without CCT) decreased at the first week postop and remained stable over the rest of the follow up, which was contrary to what was reported by Wang et al [26]. The reduction in ACD and ER in our results indicates a potential backward movement of the corneal posterior surface. This backward movement could be due to the biomechanical alternations in corneal structure caused by surgery, which would have resulted in steepening of the peripheral cornea and flattening of the central region in accordance with what was proposed by Roberts et al. [27, 28]; see Fig. 4. Furthermore, having observed a higher hyperopic shift in early postoperative stages after FS-LASIK, suggests that this procedure may induce a stronger biomechanical change than SMILE, which was considered less invasive.

A few limitations should be noted in this study. The use of different femtosecond laser in FS-LASIK and SMILE could lead to different flap and cap architectures. While it would have been better to use the same platform for both procedures, this was not possible to incorporate in Wenzhou Eye Hospital surgery routines. Another limitation was the biometric measurements taken with the Lenstar, in which patients were asked to fix their gaze on the target lamp in near distance, which may cause reflex convergence and induction of accommodation. However, these effects were mitigated as the device uses optical low-coherence reflectometry with 820 nm laser diode invisible infrared light to measure ocular biometric parameters, along with a visible fixation target designed to induce relaxation of accommodation.

## Conclusions

After laser visual correction surgery, the cornea has become slightly thicker and its posterior surface has become slightly flatter with significant posterior shift. The two procedures, FS-LASIK and SMILE, presented different effects on the ocular structure. FS-LASIK seemed to cause more pronounced topographical changes post-surgery than SMILE, possibly due to the stronger structural damage taking place in the corneal tissue in the former procedure. These observations were particularly evident in the high myopia group compared with those with low or moderate myopia. These results should help to improve the predictability of surgical outcomes and the planning and customization of future procedures.

## Data Availability

The datasets used and/or analyzed during the current study are available from the corresponding author upon reasonable request.
